# Identification of sources of resistance in cowpea mini core accessions to *Aphis craccivora* Koch (Homoptera: Aphididae) and their biochemical characterization

**DOI:** 10.1007/s10681-020-02619-5

**Published:** 2020-05-17

**Authors:** A. Togola, O. Boukar, A. Servent, S. Chamarthi, M. Tamò, C. Fatokun

**Affiliations:** 1 International Institute of Tropical Agriculture, IITA Kano Station, Kano, Nigeria; 2 CIRAD, UMR Qualisud, F-34398 Montpellier, France; 3 Qualisud, Univ Montpellier, CIRAD, Montpellier SupAgro, Université d’Avignon, Université de La Réunion, Montpellier, France; 4 International Institute of Tropical Agriculture, IITA Cotonou Station, Godomey, Republic of Benin; 5 International Institute of Tropical Agriculture, IITA-HQ Ibadan, Ibadan, Nigeria

**Keywords:** *Aphis craccivora*, *Vigna unguiculata*, Resistance mechanism, Screening techniques, Crop improvement, Biochemical compounds

## Abstract

Cowpea (*Vigna unguiculata* (L. Walp) is an important grain legume for human and livestock nutrition, especially in sub-Saharan Africa. Aphid, *Aphis craccivora* Koch (Homoptera: Aphididae), is one of the most widespread and destructive insect pests of cowpea and host-plant resistance is an effective approach to minimize the pest damage at seedling stage. This study was aimed at identifying resistant sources to *A. craccivora* within the cowpea mini core collection, a set of accessions from the largest world cowpea germplasm collection maintained at the International Institute of Tropical Agriculture (IITA). A total of 375 lines including 373 from IITA mini core collection, one resistant (TVu-801) and one susceptible (TVx-3236) checks were evaluated through artificial infestation in screening cages during the seedling stage. In cages, genotypes were planted in single rows containing four plants. They were arranged in an augmented design in which the two checks were sown in individual cages. Scoring for aphid population and damage levels were carried out on individual plants at 7, 14, and 21 days after planting. Advanced bioassays and biochemical analyses were conducted to investigate the mechanism of resistance to *A. craccivora*. Overall, three genotypes TVu-6464, TVu-1583, and TVu-15445 showed good levels of resistance comparable to the resistant check TVu-801. The HPLC analyses proved that both low sucrose levels in the plant, as well as high levels of kaempferol and quercetin, aglycones of phenolic compounds, were related with high resistance to aphids. The above genotypes with promising levels of resistance to *A. craccivora* will be used in cowpea breeding programs to develop improved resistant lines against this pest.

## Introduction

Cowpea, (*Vigna unguiculata* (L.) Walp.), is one of the most important grain legume crops for human and livestock nutrition in sub-Saharan Africa (SSA). It is cited as a major source of protein (20–32%), minerals, and vitamins, including vitamin B group, in the diet of thousands of low-income families in the region (Egho 2010; Boukar et al. 2013; Singh 2014; Togola et al. 2017). Also, its fodder is a source of quality feed for animals.

Despite its importance, cowpea production, grain yield, and quality are adversely affected by a complex of biotic and abiotic factors such as insects, weeds, diseases, drought, heat, and low soil phosphorus. Overall, insect pests are the most important limiting factors. They infest cowpea crops from the seedling stage, throughout the growing cycle, and in grains during storage. One of the most devastating and widespread insects is the cowpea aphid, *Aphis craccivora* Koch (Homoptera: Aphididae). It is the major pest affecting early stages of cowpea plants in Africa, Asia, and America (Obeng-Ofori 2007; Omoigui et al. 2017; Ouedraogo et al. 2018). The highest damage is caused by the parthenogenic apterous individuals, which are usually females. The winged form is less damaging but is responsible for the initial infestation of cowpea fields because of its ability to fly and reach new fields. The adults and nymphs feed on the under-surface of young leaves, stem tissues, growing tips, petioles, flowers, and pods of mature plants by sucking the fluid (Togola et al. 2017). Attack by aphid results in stunting, leaf distortion, premature defoliation, and death of seedlings, the most susceptible developmental stage of cowpea. Indeed, aphids are piercing-sucking insects that feed on a plant’s phloem sap, which is essential for plant growth (Dixon 2012; Soffan and Aldawood 2014). According to Dixon 2012, the sap ingested by aphids consists mainly of a concentrated solution of simple sugars and a weak solution of amino acids. Adult aphids process at least their own weight of phloem sap per day while the immature nymph processes several times their weight. Also, aphids secrete honeydew, leading to mold formation, thereby reducing photosynthetic efficiency of the plant (Singh and Jackai 1985; Annan et al. 1985; Aliyu and Ishiyaku 2013; Huynh et al. 2015). In addition to the direct feeding damage, *A. craccivora* transmits at least 14 viral diseases (Thottappilly et al. 1990), of which the cowpea aphid-borne mosaic virus is the most devastating (Bata et al. 1987; Blackman and Eastop 2000; Kusi et al. 2010; Yang et al. 2015). Aphid infestations are particularly severe during dry spells (Jackai and Daoust (1986), especially if the seedling stage is affected (Souleymane et al. 2013; Huynh et al. 2015). The induced cowpea yield losses can reach or exceed 50% in the case of high and uncontrolled aphid infestation or in the case of legume virus infection even at low population densities (Obopile and Ositile 2010). Cowpea aphid attacks a wide range of plant species but prefers those of the Fabaceae Family (beans, peas, groundnuts).

In sub-Saharan Africa, farmers often rely on foliage spraying using synthetic pesticides to prevent initial infestation by aphids. Despite their efficacy, these chemicals can be noxious to humans and environmental health and affect the activity of beneficial insects (Souleymane et al. 2013). Host-plant resistance remains one of the most effective approaches to minimizing aphid damage on cowpea and many other crops (Huynh et al. 2013; Smith and Chuang 2014; Huynh et al. 2015). In past and recent studies, several cowpea lines were evaluated for resistance to *A. craccivora* but the types and roles of plant biochemicals involved in such resistance were not well elucidated. For instance, the role of plant sugar contents in the resistance of various crops to aphid species was reported (Mittler 1967; Kennedy and Schaefers 1975; Farrell 1977; Corcuera 1993; Quiros et al. 1977) but a specific content was not indicated regarding the resistance mechanism in cowpea. Similarly, some studies have discovered the role of an array of plant phenolic or flavonoid contents in the resistance of cowpea to aphids (Ofuya 1997; Lattanzio et al. 2000).

This study was aimed at identifying the resistant genotypes within the IITA cowpea mini core collection to *A. craccivora* and determine the metabolites that mediate such resistance. The identified resistant/tolerant genotypes will be used in the cowpea breeding program to improve the crop’s productivity in aphid endemic areas.

## Materials and methods

### Cowpea genotypes

A total of 375 cowpea genotypes including 373 accessions from the International Institute of Tropical Agriculture (IITA) mini core collection and two checks were used in the experiments. Accession TVu-801 was used as resistant check because of its good resistance to *A. craccivora* (Ofuya 1997; Togola et al. 2017) while variety TVx-3236 was the susceptible check (Bata et al. 1987; Souleymane et al. 2013). Only untreated and clean seeds of the test entries and checks were used in this experiment. The 373 mini-core genotypes are known to have desired genetic traits including good grain yields, farmer-preferred seed colors and sizes, and short-to-medium maturing duration. None of the 373 genotypes were screened before for resistance to *A. craccivira*, therefore their level of resistance was not known.

### Aphid cultures

Aphid cultures were initiated by sampling a single colony of *A. craccivora* adults from cowpea fields at the IITA Minjibir Research Farm, Latitude 12° 14′ 35.30″ N and Longitude 8° 66′ 62.10″ E located at about 45 km from Kano City (Kano State, Nigeria). The sampling was done on the beginning of the rainy season where the aphid population is high in cowpea fields. The aphid cultures were maintained in insect-proof cages to protect them from predators and parasitoids’ attack. The rearing was carried out on 2-week-old seedlings of the susceptible cowpea variety TVx-3236, planted fortnightly in new cages to ensure continuous availability of aphids throughout the period of the study. Only the fourth-instar nymphs of the insect were used to infest cowpea seedlings during the screening cage and laboratory experiments. Enough colony was maintained in the same environment in order to infest all of the seedling same days with the same population of fourth-instar aphid nymphs.

### Artificial screening of cowpea genotypes

This experiment was conducted using a validated artificial screening cages method (Singh and Jackai 1985) in which test entries and checks were initially and randomly planted in wooden trays filled with soil (two-thirds of top soil plus one-third of compost) and kept in insect-proof cages. Each cage had two trays of 40 cm width, 40 cm length, and 11 cm height and each tray was planted with five entries. So, ten entries were planted in each cage where seeds of individual genotypes were sown in single rows of four hills 10 cm apart, making four plants per entry. The genotypes were arranged in an augmented design in which the two checks were sown in individual cages. Irrigation was performed once a day to avoid any water stress. Good seeds (well-formed and without damage or disease symptoms) were used in order to get uniform emergence and age of seedlings. At 7 days after sowing, individual seedlings of each variety were separately infested with ten fourth-instar nymphs using a camel-hair brush (Jackai and Singh 1988; Souleymane et al. 2013). All aphids used in the experiment were collected from the same culture to avoid dealing with multiple biotypes. Aphid populations (pop) as well as damage to seedlings (DS) were scored visually at 7, 14, and 21 days after infestation (DAI). The experiment was maintained until the death of the susceptible check (TVx-3236). A second experiment was conducted for confirming the resistance status of the identified resistant entries from the initial test. The same screening facilities and methods were used, but five hills of each entry were considered instead of four. This experiment went beyond 21 days after infestation to allow assessing the number of dead plants (DP) and number of days to plant death (DTD). Eight accessions including the three most resistant genotypes obtained from this second test, two moderately resistant, one highly susceptible genotype and the two checks, were used in the aphid feeding bioassay and also for the biochemical characterization.

As described below, aphid population levels were assessed using a 1–9 visual rating scale where 1 indicates few or no aphids (0–4 aphids), 3 relates to few isolated colonies (5–20 aphids), 5 to several small colonies (21–100 aphids), 7 to large isolated colonies (101–500 aphids), and 9 to large continuous colonies (more than 500 aphids per seedling). Damage severity was scored using a 1–5 scale where 1 and 2 indicate a good level of resistance, 3 moderate resistance, 4 moderate susceptibility, and 5 high susceptibility (Jackai and Singh 1988).

### Laboratory bioassay

The eight accessions selected for the laboratory bioassays comprised three genotypes having the highest levels of resistance in the previous screening experiment (TVu-15445, TVu-1583, TVu-6464), two genotypes among those that showed moderate resistance (TVu-415 and TVu-467), one genotype from those that displayed high susceptibility (TVu-1727) and the two checks (TVx-3236 and TVu-801). These selected genotypes were subjected to detailed bioassays in order to understand their antibiotic effects on *A. craccivora*. For this purpose, three trifoliate leaflets (1 leaf) were cut from 15-day-old seedlings of each selected genotype and checks, put in small plastic jar of 50 ml, infested with three pre-reproductive wingless adult aphids, and kept in the laboratory at IITA, Kano Station. The jars were arranged in a completely randomized design with four replicates per test entry. The experiment was monitored every 2 days during which the old leaves were replaced with new ones. Also, aphid population growth parameters (including new progeny, survival, and mortality) were recorded. The experiment lasted for a period of 7 days.

### Biochemical characterization

Biochemical analysis was performed using the laboratory analysis protocol of UMR-Qualisud at Montpellier, France, in order to establish the mechanisms conferring aphid resistance to the eight selected genotypes identified as resistant (TVu-15445, TVu-1583, TVu-6464, and TVu-801), moderately resistant (TVu-415 and TVu-467), or susceptible (TVx-3236, TVu-1727). All chemicals used were bought from Sigma-Aldrich (St. Louis, MO, USA) and presented a HPLC purity. The investigation was limited to the essential compounds involved in plant resistance to *Aphis craccivora* according to literature review.

#### Sample collection and treatment

Fresh biomass of 15-day-old cowpea seedling was collected and oven-dried at 45 °C for 72 h at IITA Kano Station, and the dried samples were sent to UMR-Qualisud, Montpellier, France, for biochemical characterization. For quality assurance purposes, samples were coded and one check was duplicated prior to being couriered to France.

#### Dry matter measurement

Before analysis, samples were crushed and ground to a powder using a Seb laboratory knife grinder (Ecully, France). The total residual dry matter (DM) was measured in an oven under vacuum at 30 mbar and 70 °C during 48 h according to AOAC procedures (AOAC 1990). All DM were carried out in triplicate.

#### Total sugar measurement

In order to extract soluble carbohydrates, 0.5 g of sample was mixed with 80% ethanol in a ratio 1/30 (sample/solvent, g/ml) at 70 °C for 15 min under agitation. The extract was cooled and centrifuged for 10 min at 10,000 × *g* and 10 °C. The supernatant was recovered and residues were extracted two more times using the same procedure. Supernatants were pooled and filtered through a 0.45-μm syringe filter prior to HPLC analysis.

Glucose, fructose, sucrose, and lactose contents were determined by HPLC using a Dionex Ultimate 3000 system (Dionex, USA) equipped with corona detector (electrospray) and a diode array detector. A Shodex Asahipak NH2P-50 of 250 mm × 4.6 mm × 5 μm (Shodex, Japan) with a mobile phase composed of pure water (phase A) and pure acetonitrile (phase B) was used. Setting was done with an isocratic elution program (30% phase B, 70% phase A) at a flow rate of 1 ml min^−1^ and a column temperature of 30 °C. Injection volume was set at 20 μl and spectrophotometric detection was set at 210 nm. Calibration curves were calculated using base 10 logarithm. All analyses were realized in triplicate.

#### Measurement of polyphenols

Polyphenol extractions and quantifications were realized as described in some past research (Cai et al. 2003; Chen et al. 2015) with slight modifications. Free polyphenols were extracted as follows: 800 mg of dried sample was mixed in 2 ml of water. Methanol was added in order to reach a concentration of 80% of methanol in a ratio 1/10 (sample/solvent, g/ml) and the mixture agitated during 1 h under nitrogen atmosphere in order to avoid oxidation. After extraction, the mixture was centrifuged at 10,000 × *g* for 10 min at 10 °C. The supernatant was recovered and the residues extracted twice with 80% methanol. Supernatants were pooled and injected in HPLC after 0.45-μM syringe filtration. In order to analyze bound polyphenols, free polyphenol extractions were dried for one night at 40 °C in a ventilated oven and 500 mg of dried residue was hydrolyzed in 5 M NaOH for a ratio 1/14 during 24 h under nitrogen atmosphere and continuous agitation. The pH was adjusted to 2.0 using 12 N HCl. The mixture was fractionated using diethyl ether-ethyl acetate (1/1 v/v) and agitated for 20 min. After centrifugation (10,000 × *g*, during 10 min and at 10 °C), the supernatant was recovered and the residues extracted twice with 10 ml of diethyl ether/ethyl acetate. All the organic phases were mixed and evaporated to dryness.

The final residue was recovered in 80% methanol and was injected in HPLC following filtration with 0.45-μM syringe. Aglycones of bound and free polyphenols were analyzed after alkaline hydrolysis. The extract was mixed with NaOH 5 M in a ratio 1/5 and agitated for 10 h under nitrogen atmosphere. Then, pH was adjusted to 2.0 using 12 N HCl. The extract was then diluted in 80% methanol and filtered through 0.45-μM syringe prior to HPLC analysis. Agilent 1200 chromatography (Agilent, USA) equipped with a diode array detector was used for analysis. A volume of 20–100 μl was injected through a C18 ACE 250 mm × 4.6 mm × 5 μM column (Advanced Chromatography Technologies Ltd, Scotland). DAD was set at 280, 330, and 380 nm. Mobile phases were 1% formic acid in pure water as phase A and acetonitrile as phase B. Flow was set at 0.7 ml min^−1^ and at 30 °C. Gradient was fixed at 98% of A and 2% of B (at initial stage), stabilized at 2% B for 10 min, increasing at 20% of B from 10 to 30 min, to 40% B from 30 to 50 min, to 60% B from 50 to 70 min, to 80% B from 70 to 80 min, to 100% B from 80 to 90 min, returned to initial condition (2% B) in 5 min and maintained for 10 min.

Compounds were identified based on their retention times, their UV–Vis spectra, and their mass spectra. Mass spectrum was acquired using a Synapt G2-S (Waters, USA) set at ESI- ionization, for a range of mass of 50–1600 Da, with a source at 140 °C, a capillary tension of 3 kV and a desolvation temperature of 450 °C with the same chromatographic parameters. All quantifications were performed in triplicate.

We were not able to do a “total polyphenols” (PPT) analysis by colorimetry. However, each phenol separated by HPLC e.g., Kaempferol has been quantified based on the calibration curve to get its total.

### Data analysis

Means of non-parametric data (e.g., population and damage scores) were calculated using Excel and accessions were classified per resistant category based on means using the scale described by Jackai and Singh (1988). Other phenotyping data such as number of dead plants, days to plant death, emerged aphid progeny, total aphid population, dead aphid population, and mortality rate collected from screen cages and laboratory experiments were subjected to analysis of variance using SAS 9.4 to determine if there were significant differences among the cowpea genotypes. The LSD test was used to separate the means. Breeding View software was used to establish the correlations between five means parameters, namely aphid damage score at 21DAI, the number of survived plants, the emerged aphid progeny from the bioassay experiment, the mortality rate from the bioassay experiment, and plant sucrose content.

## Results

### Cowpea phenotyping for resistance to *Aphis craccivora* in screening cage

Results of the initial test showed different levels of resistance among the test entries at 7, 14, and 21 days after infestation in which the level of resistance was plant dependent.

At 7 days after infestation, only 49 genotypes including the susceptible check TVx-3236 obtained a maximum population score of 7. At the same period, the maximum damage score obtained by 21 genotypes was 3.

At 14 days after infestation, 128 genotypes got the highest population score (e.g., 9), and 215 entries recorded a population score of 7. At the same period, the maximum damage score (e.g., 9) was recorded by eight genotypes, the most susceptible among the test entries, while majority of the genotypes (e.g., 204) had a damage score of 3. Genotype TVu-6464 was the only entry that did not show any damage symptoms at this period (Table 1).

**Table 1:** Variation of aphid population and damage on cowpea seedlings at 7 and 14 days after artificial infestation in screening cages using ten aphid nymphs per plant

Measurement of aphid population			Measurement of aphid damage on seedlings		
Population score	Number of infested genotypes at 7 DAI	Number of infested genotypes at 14 DAI	Damage score	Number of damaged genotypes at 7 DAI	Number of damaged genotypes at 14 DAI
1	0	0	1	268	1 (TVu-6464)
3	109	10	2	81	67
5	212	17	3	21	204
7	49	215	4	0	90
9	0	128	5	0	8

**Fig. 1:**
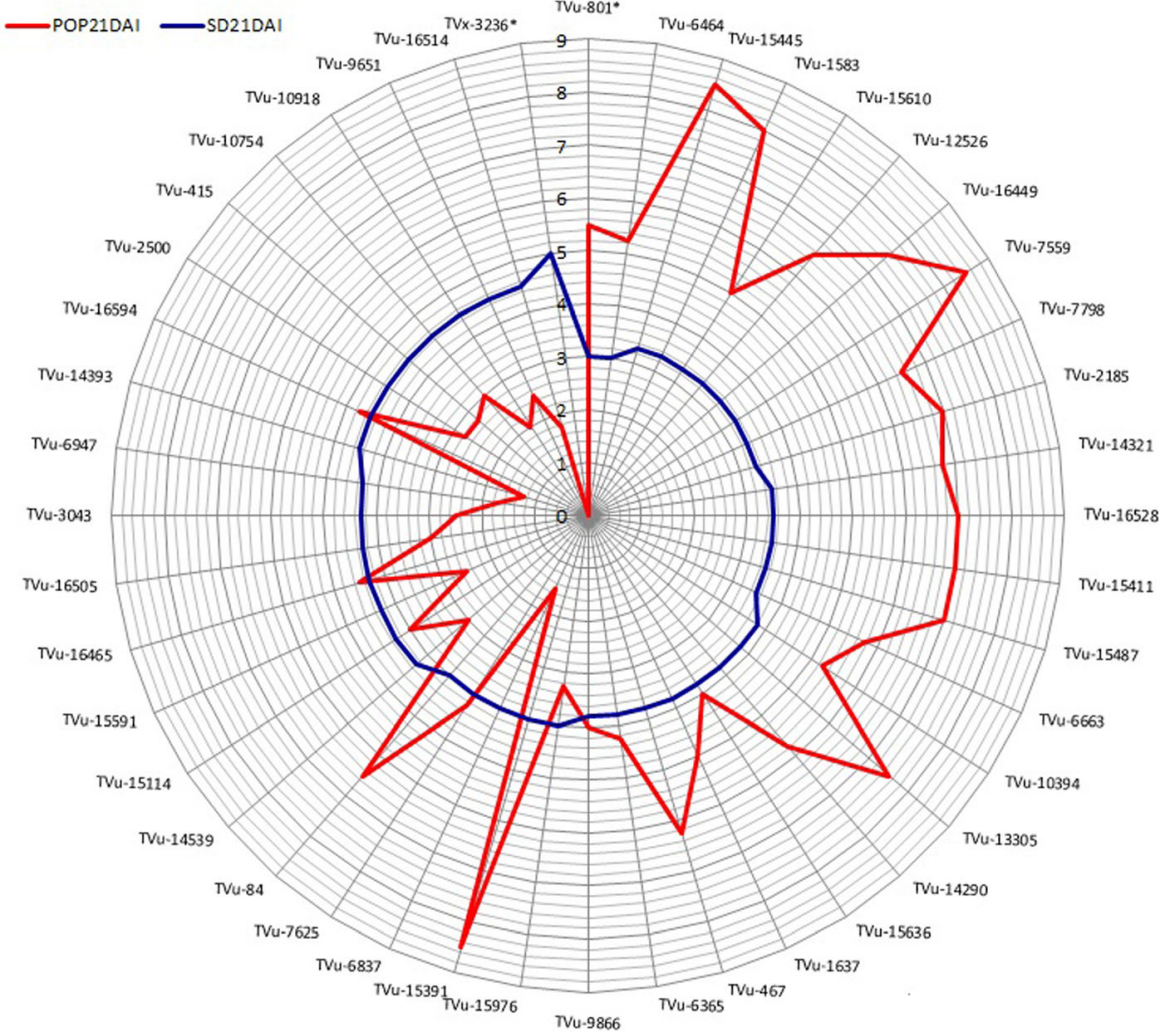
Aphid population and damage scores on the moderate and good resistant genotypes at 21 days after infestation from the initial evaluation in screening cages (*Resistant and susceptible checks)

At 21 DAI, nine mini-core genotypes (TVu-6464, TVu-15445, TVu-1583, TVu-15610, TVu-12526, TVu-16449, TVu-7559, TVu-7798, and TVu-2185) showed significantly low damage by aphid as well as the resistant check TVu-801, while 25 genotypes showed moderate aphid damage with scores between 3.3 and 4.3. Also, nine genotypes were alive (green) despite high damage score (4.5) (Fig. 1). Accessions TVu-15391 recorded the highest population while still alive. All the remaining mini-core genotypes were severely damaged by aphids, as they completely wilted or died. At this stage, the aphid population was higher on surviving genotypes as individuals had migrated from the wilted and dead plants (Fig. 1).

**Fig. 2:**
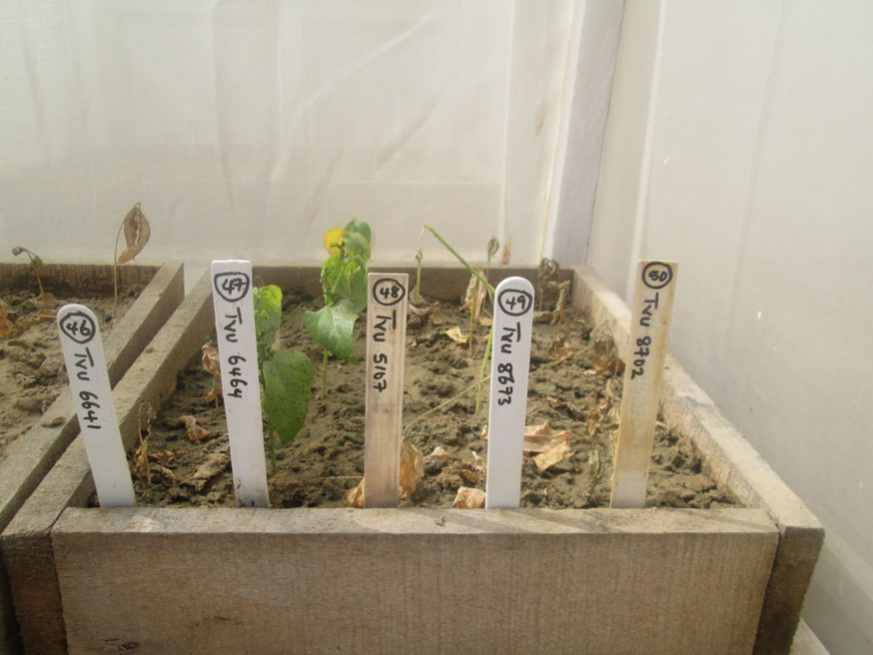
Genotype TVu-6464 showing good resistance to *A. craccivora* attack

**Fig. 3:**
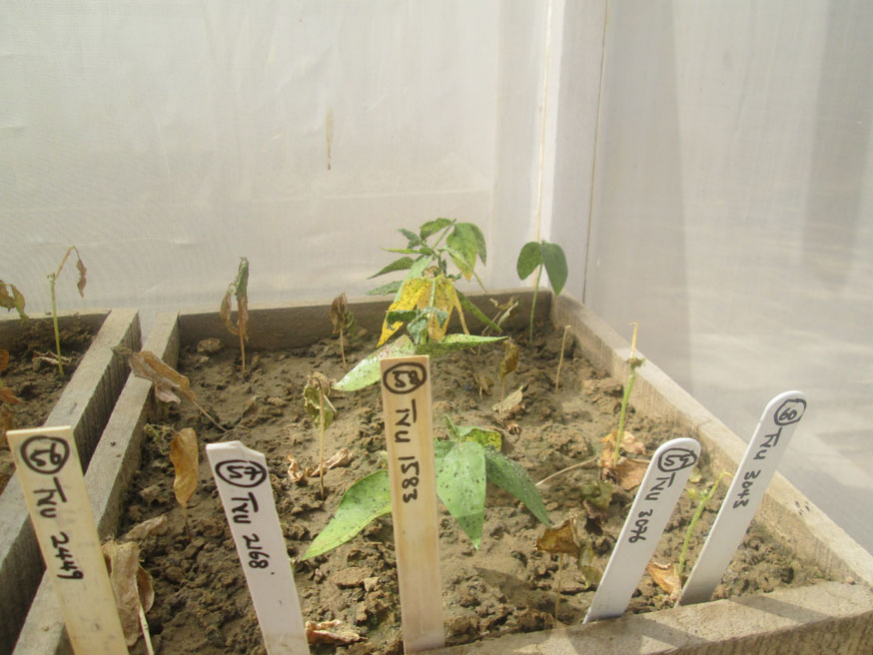
Genotype TVu-1583 showing good resistance to *A. craccivora* attack

**Fig. 4:**
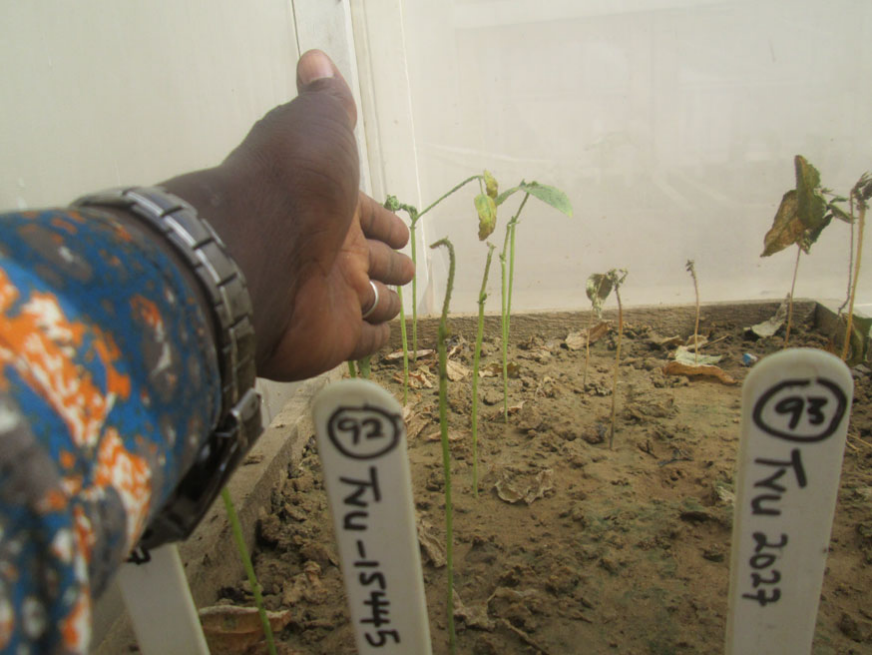
Genotype TVu-15445 showing good resistance to *A. craccivora* attack

**Fig. 5:**
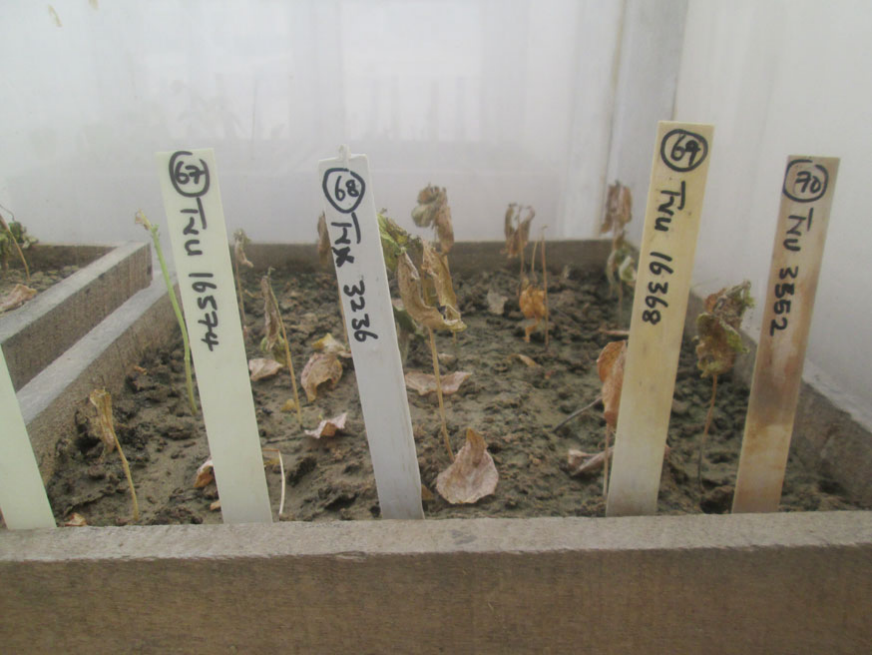
Susceptible check TVx-3236

From the confirmation test, three mini-core genotypes—TVu-6464, TVu-1583, and TVu-15445 as well as the resistant check TVu-801 recorded the lowest damage scores (2.0–2.3) at 21 days after infestation and they stayed green until the end of the experiment at 40 days after infestation (Table 2, Figs. 2, 3, 4, 5). These genotypes can be classified as resistant to *A. craccivora*. Also, 18 genotypes including TVu-467 and TVu-415 showed moderate seedling damage scores (3.2–3.4) at 21 days after infestation and two- to three-fifths of their seedlings stayed green for 25–33 days after infestation. They can be classified as moderately resistance to *A. craccivora* (Table 2). The remaining mini-core genotypes including TVu-1727 and TVx-3236 were susceptible to *A. craccivora* as their seedlings wilted or died within 21 days after infestation.

**Table 2:** Genotypes showing significantly low damage as compared to the susceptible checks at 21 days after infestation in screen cages at Kano

Genotypes	POP21DAI	SD21DAI	Dead or wilted plants	Number of surviving plants	DTD	Final status
TVu-801*	6.0	2	0	5	40**	R
TVu-6464	6.0	2	0	5	40**	R
TVu-15445	7.0	2.3	0	5	40**	R
TVu-1583	7.0	2.3	0	5	40**	R
TVu-10754	7.0	3.2	2	3	27.5	MR
TVu-7559	8.5	3.3	2	3	33.3	MR
TVu-7798	6.5	3.3	2	3	30.3	MR
TVu-15610	5.0	3.3	2	3	32.8	MR
TVu-12526	6.0	3.3	2	3	32.5	MR
TVu-16449	7.25	3.3	2	3	27	MR
TVu-6837	2.0	3.3	3	2	25.8	MR
TVu-10918	7.0	3.3	3	2	26.5	MR
TVu-16514	6.0	3.3	3	2	26	MR
TVu-2185	6.5	3.3	3	2	25.6	MR
TVu-14321	6.5	3.3	3	2	33.5	MR
TVu-6365	4.25	3.3	3	2	25.8	MR
TVu-6663	5.75	3.3	3	2	25.8	MR
TVu-15636	4.0	3.3	3	2	25.8	MR
TVu-467	6.0	3.3	3	2	28.3	MR
TVu-415	3.5	3.3	3	2	24.5	MR
TVu-3736	6.5	3.4	3	2	26	MR
TVu-15391	7.5	3.4	3	2	25.7	MR
TVx-3236*	0.5	5.0	5	0	20.3	S
TVu-1727	0.0	5.0	5	0	16.0	S

Values shown in this table represent the means of the measured parameters. POP21DAI = insect population at 21 days after infestation; SD21DAI = score of damage at 21 days after infestation; DTD = days to seedling death

*Checks, **Date to end of experiment

### Laboratory bioassay

The feeding behavior of aphids in laboratory bioassay showed that some reproductive parameters such as the number of emerged progenies, total aphid population, and mortality rate differed significantly among the test entries. The emerged progenies and total aphid population obtained after 7 days of feeding were significantly lower on genotypes TVu-6464 and TVu-15445 compared to the susceptible check TVx-3236. As for the mortality rate, highly significant differences were noted between the entries. Among the test entries, the highest mortality rate was recorded from TVu-6464 (95.2) followed by TVu-15445 (93.8%) and TVu-1583 (93.5%). Their mortality rates were in the same range as that of the resistant check TVu-801 (96.1%). In contrast, the mortality rate in the susceptible TVx-3236 was the lowest (58.8%) (Table 3).

**Table 3:** Aphid population dynamics following laboratory bioassay

Genotype	Emerged progenies	Dead population	Mortality rate
TVu-15445	12.0	13.5	93.8
TVu-1583	20.3	22.0	93.5
TVu-1727	28.0	22.3	71.7
TVu-415	28.7	24.3	76.8
TVu-467	32.7	23.0	64.8
TVu-6464	8.0	10.7	95.2
TVu-801*	28.3	30.3	96.1
TVx-3236*	40.0	26.0	58.8
*p* value	0.025	0.632	0.001
Mean LSD	25.6	21.5	16.7

*Checks; *DAI* days after infestation

### Biochemical characterization

#### Sugar content in dried matter

The results of biochemical analyses showed that sucrose and fructose were the dominant sugar compounds in the cowpea leaf samples (46.75 and 34.37%, respectively). Sucrose concentration was most variable among the sugars in the selected test genotypes. Sugar content in general, and especially sucrose, appeared higher in aphid-susceptible genotypes than in aphid-resistant ones (Table 4). For instance, genotype TVu-1727 tested as the most susceptible genotype to aphid, as it showed the highest content of total sugar (53.17 g kg^−1^) and the highest sucrose content (35.08 g kg^−1^). The susceptible TVx-3236 also showed high content of total sugar (29.64 g kg^−1^) and sucrose (15.1). Genotypes TVu-6464 and TVu-15445 tested as resistant to aphid recorded low content of total sugar (21.63 g kg^−1^ and 24.63 g kg^−1^, respectively) and sucrose (7.67 g kg^−1^, 10.03 g kg^−1^, respectively). Unexpectedly, accession TVu-1583, which tested resistant to aphids, recorded high sucrose and total sugar contents while the resistant check TVu-801 recorded the lowest values of both total sugar (18.07 g kg^1^) and sucrose (3.73 g kg^−1^) (Table 4).

**Table 4:** Sugar content in selected mini-core accession (in g kg^−1^ dried matter)

Sample	Fructose	Glucose	Sucrose	Lactose	Total sugar
TVu-1583	13.24 ± 0.78	2.47 ± 0.05	17.57 ± 0.72	3.35 ± 0.16	36.63
TVu-6464	8.96 ± 0.33	0.89 ± 0.05	7.67 ± 0.11	4.13 ± 0.21	21.65
TVu-15445	10.34 ± 0.56	0.86 ± 0.09	10.03 ± 1.05	3.4 ± 0.55	24.63
TVu-467	11.42 ± 0.07	2.27 ± 0.04	14.49 ± 0.78	3.33 ± 0.27	31.51
TVu-415	7.84 ± 0.23	1.15 ± 0.08	6.74 ± 0.12	5.13 ± 023	20.86
TVu-1727	11.32 ± 1.25	3.55 ± 0.58	35.08 ± 2.24	3.22 ± 0.68	53.17
TVu-801^®^	9.86 ± 0.49	1.61 ± 0.13	3.73 ± 0.36	2.87 ± 0.17	18.07
TVx-3236*	8.19 ± 0.58	1.05 ± 0.06	15.1 ± 1.15	5.3 ± 0.46	29.64

Values shown in this table are average sugar content ± SD) quantified by HPLC. ^®^Resistant check, *Susceptible check

**Fig. 6:**
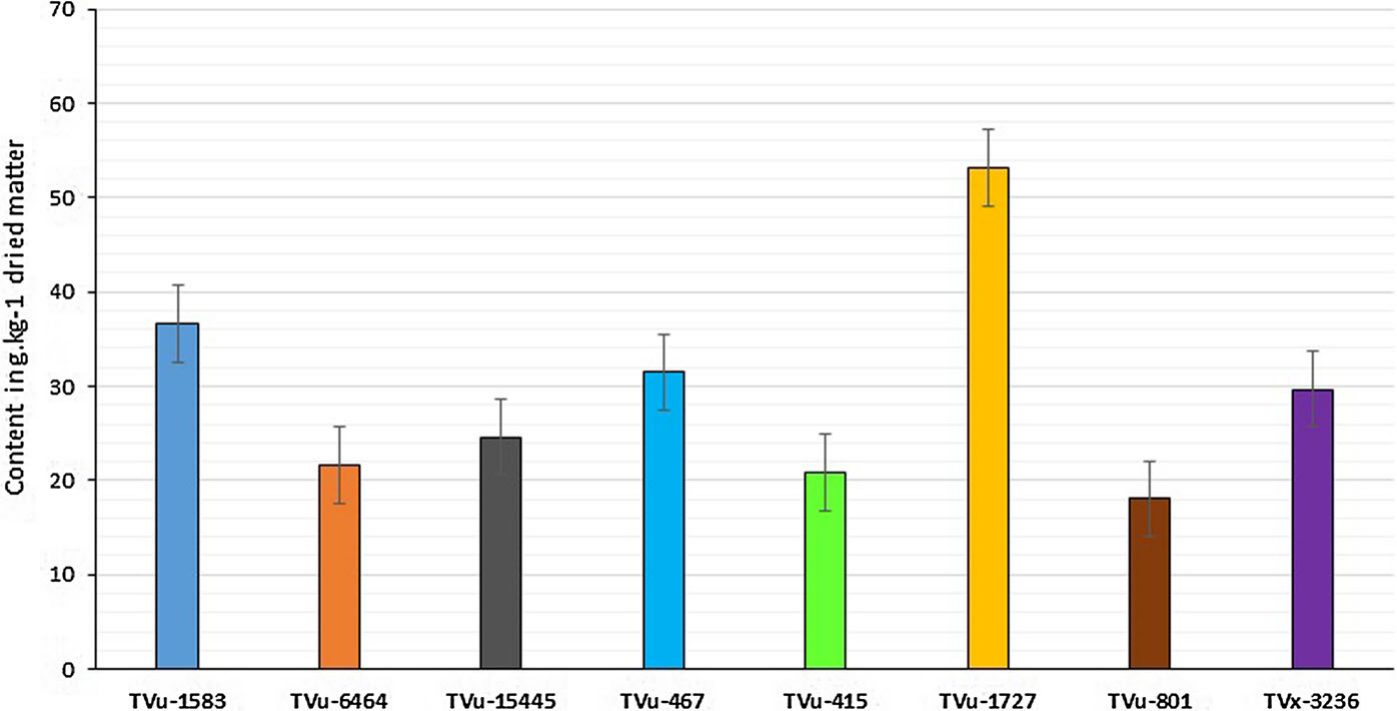
Total sugar content of selected genotypes in g kg^−1^ dried matter (*error bars* are the standard error of the means)

**Fig. 7:**
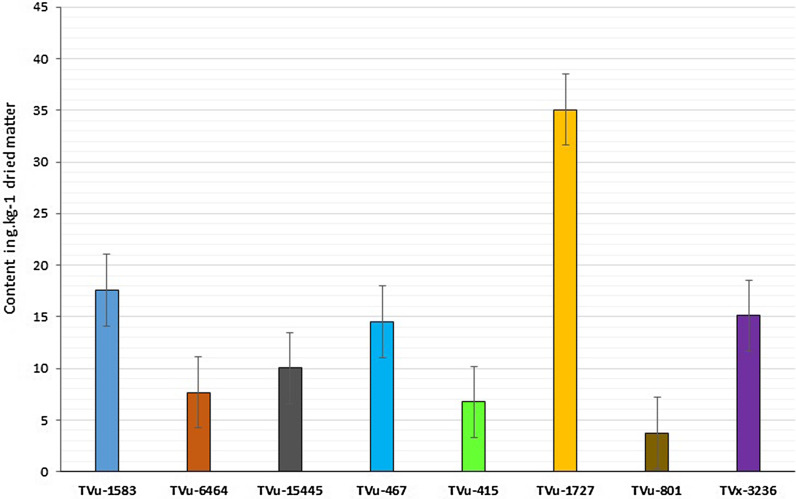
Sucrose content of selected genotypes in g kg^−1^ dried matter (*error bars* are the standard error of the means)

Sucrose content in samples followed the same trends as the total sugar content (Figs. 6 and 7). Therefore, sucrose appeared to be the main sugar component influencing the susceptibility of cowpea to *Aphis craccivora*.

A significant and positive correlation (*P* < 0.001, *r* = 0.665) was found between damage score at 21 DAI and sucrose content in the plants while a negative and no significant correlation was noted between the aphid mortality rate and sucrose content (*P* > 0.05; *r* = − 0.487). Similarly, no significant correlation was noted between aphid emergence rate and sucrose content (*P* > 0.05; *r* = 0.112) (Table 5).

**Table 5:** Correlations between some parameters in cowpea genotypes under *A. craccivora* infestation

Measured parameters	1	2	3	4	5	6
1. SD21DAI	–					
2. Survived plants	− 0.953***	–				
3. Emerged aphid progeny at 7 DAI	0.614*	− 0.621*	–			
4. Mortality rate	− 0.933***	0.979***	− 0.731***	–		
5. Sucrose content	0.665*	− 0.520*	0.112 ^ns^	− 0.487 ^ns^	–	
6. Total sugar content	0.5570*	− 0.411^ns^	0.079^ns^	− 0.391^ns^	0.983	–

*(Significant with *P* < 0. 05); ***(Highly significant with (*P* < 0.001); ^ns^(Not significant)

#### Mass identification of main free phenolic compounds in cowpea samples by HPLC–MS

**Fig. 8:**
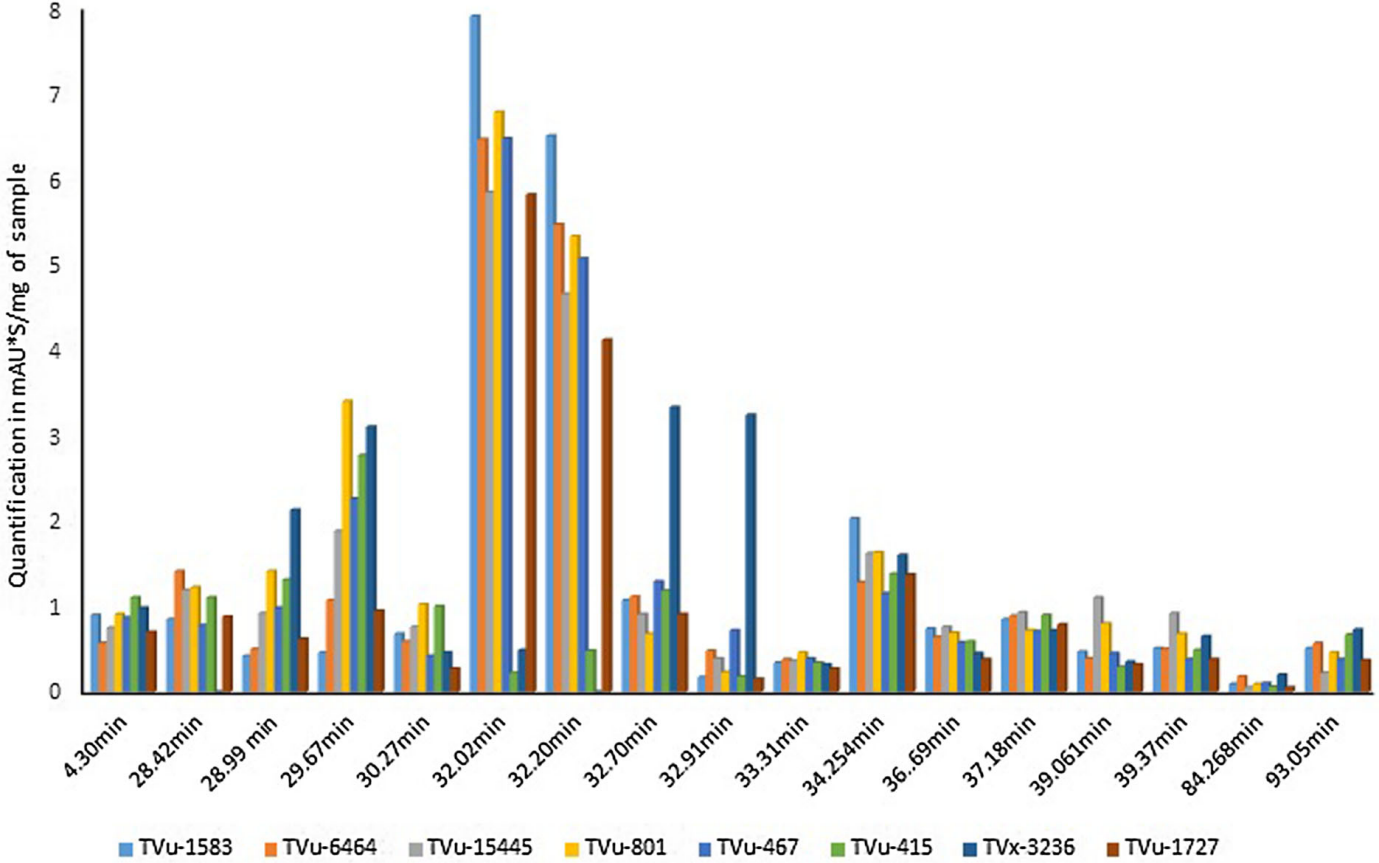
Seventeen peaks of unknown phenolic compounds found in cowpea samples represented by area 330 nm/mg of each sample

HPLC analysis showed 17 polyphenols peaks but only seven peaks appeared to be more discriminant (Fig. 8). The main free phenolic compounds were detected at Peak 6 (RT 32.02) and Peak 7 (RT 32.20) where the tested cowpea samples exhibited various intensities of absorbance. In opposite to sugar content in cowpea, phenolic compound appeared to be in higher concentration in aphid-resistant genotypes than in aphid-susceptible ones (Fig. 8). This clearly shows a correlation between polyphenol content and pest resistance. Genotypes TVu-1583, TVu-6464, TVu-15445, and the resistant check TVu-801 showed the highest free phenolic proportion at both Peaks 6 and 7. Also, the moderately resistant genotype TVu-467 showed high free phenolic proportion while the susceptible TVx-3236 showed the lowest free phenolic proportion (Fig. 8).

Bound polyphenol contents were very low compared to free polyphenols. Moreover, there was no difference in the polyphenol content between the samples. Therefore, in cowpea, all the phenolic main compounds useful to understand host-plant resistance did not originate from the cell wall or linked by fibers.

Mass identification of main phenolic compounds from free extraction detected kaempferol and quercetin as the main aglycone playing significant difference between cowpea samples during retention times 32.02 min and 32.20 min (Table 6). Thus, these metabolites were mainly associated with host-plant resistance to *Aphis craccivora* in cowpea. Aglycones types and contents found during the remaining retention times did not vary significantly between samples.

**Table 6:** Mass identification of main free phenolic compounds

Retention time (min)	Mass-to-charge ratio (*m/z*) M–H	Raw formula	Identification of raw formula basis
28.42	355.1	C16H20O9	1-*O*-Feruloyl-beta-d-glucose
28.99	613.08	C28H22O16	Kaempferol 3-*O*-(2″-*O*-galloyl)-glucuronide
29.67	613.08	C28H22O16	Kaempferol 3-*O*-(2″-*O*-galloyl)-glucuronide
30.27	757.18	C32H38O21	Delphinidin 3-sambubioside 5-glucoside
32.02	1251.29	C61H56O29	Quercetin 3.7-diglucoside
	921.19	C40H42O25	
	625.14	C27H30O17	
32.20	931.11	C39H32O27	Kaempferol 3-*O*-(2″-*O*-galloyl)-glucuronide
	613.18	C28H22O16	
32.70	903.22	C41H44O23	Calabricoside B
32.91	463.09	C28H16O7	
33.31	463.09	C32H16O4	
34.25	933.23	C42H46O24	Kaempferol 3-(2′″-(E)-caffeylsophoroside)-7- glucoside
37.18	581.09	C28H22O14	Cyanidin 3-[6-(6-*p*-hydroxybenzoylglucosyl)-2- xylosylgalactoside]
	861.21	C13H42O22	
39.06	771.18	C36H36O19	Kaempferol 3-caffeylsophoroside
39.37	801.19	C37H38O20	Quercetin 3-(2′″-feruloylsophoroside

## Discussion

Results from the screening showed that none of the test entries had a damage score exceeding 3 at 7 DAI. Also, the majority of the test entries did not show any damage symptoms. However, 102 entries exhibited initial aphid damage symptoms at this period. This result demonstrated that only susceptible entries show symptoms of aphid damage at 7 DAI while moderately resistant and resistant genotypes did not. Further, the result demonstrated that the majority of the mini-core accessions were not susceptible at this very early stage (7 DAI).

At 14 days after infestation (14 DAI), most of the mini-core accessions recorded high population density of aphid. At this stage, significant discrimination between genotypes was noted. Eight genotypes had high aphid infestation and were severely damaged. This means that 14 DAI is a more relevant period for investigating the resistance to *A. craccivora* on cowpea seedling than 7 DAI.

At 21 DAI, differences in damage symptom expressions on resistant, moderately resistant, and susceptible genotypes were most apparent. The resistant genotypes appeared green or carried few damage symptoms while the susceptible genotypes were wilted, yellow, or dead. As for insect population score at this stage, the highest densities were recorded on the resistant and moderately resistant genotypes, while the lowest densities were noted on susceptible genotypes that were wilted or died. This unexpected presence of high population density on the resistant genotypes should be considered as a temporary circumstance where aphids had moved from the wilted and dead plants (mostly the susceptible ones) to feed on the fresh and green plants (mostly the resistant ones) that were only available during that period. Therefore, the aphid population at 21 DAI cannot be evidence of host susceptibility in all cases, especially in situations of no-choice. In all experiments, the susceptible check Tvx-3236 died before 21 days after infestation. It can be concluded that aphid-susceptible genotypes do not survive more than 21 days under high aphid infestation. Therefore, 21 DAI can be considered as a suitable period to determine the resistance status of cowpea seedling to *A. craccivora*, especially when the susceptible check had died by this time. However, genotypes considered resistant at 21 DAI should be evaluated until 28 DAI or beyond to monitor the variation of pest population.

Overall, results from artificial screening showed low aphid damage on mini-core genotypes TVu-6464, TVu-1583, and TVu-15445, as well as the resistant check TVu-801. The ability to withstand aphid attacks by these genotypes throughout the series of initial and confirmation experiments demonstrates their good resistance level to aphids. Indeed, TVu-6464 and TVu-1583 remained green during the whole experiment, while TVu-15445 responded to aphid attacks by losing leaves while maintaining the stem green before recovering totally. Genotypes TVu-467 and TVu-415 were found to be moderately resistant to aphid damage in this study. Both genotypes can survive, grow, and produce well in conditions where the aphid population is not too high. Genotype TVu-1727 was as susceptible as the susceptible check TVx-3236. The feeding behavior of aphids in laboratory bioassay showed that the aphid mortality rate was significantly higher in TVu-6464, TVu-15445, and TVu-1583 than in susceptible check TVx-3236. Also, the number of emerged progenies and total aphid population were significantly lower in resistant genotypes compared to the susceptible check. The adverse effects of feeding on leaves of genotypes TVu-6464, TVu-15445, TVu-1583, as well as TVu-801 on the reproductive parameters of *A. craccivora* indicate that antibiosis was the basis of their aphid resistance. The antibiotic capacity in resistant cowpea can slow down the development of aphid population (Teetes 2007; Alabi et al. 2012; Omoigui et al. 2017). Thus, feeding on the leaves of genotype TVu-6464 resulted in the lowest emerged progeny and 95.2% mortality. This genotype was the least favorable to aphid multiplication. The antibiotic activity pairs with high mortality rate or reduced longevity (Teetes 2007). Past research has revealed that the antibiosis in cowpea can be attributed to phenolic content (Ofuya 1997; Togola et al. 2017).

In this research work, the biochemical characterization revealed that sucrose and fructose were the dominant sugar components in the mini-core cowpea seedlings in general but sucrose content appeared to be significantly higher in aphid-susceptible genotypes than in aphid-resistant ones. The results showed a clear relationship between cowpea susceptibility to aphid (high damage) and high level of sucrose in seedlings (*r* = 0.665). This indicates a significant role of sucrose in aphid feeding activity. Indeed, the level of sucrose was low in the moderately resistant and resistant accessions (except in TVu-1583) and significantly high in the susceptible ones. The levels of the other sugar compounds (fructose, glucose, and lactose) were more stable in both resistant and susceptible accessions. The low level of sucrose in the resistant mini-core genotypes has surely played a big role in their resistance to *A. craccivora*. Several past studies have reported the role of sucrose in nutrient uptake by aphid species and that fluid uptake by the green peach aphid *Myzus persicae* Sulzer (Homoptera: Aphididae) was poor or non-existent on diets having low sucrose (Mittler 1967). This is in agreement with other findings which indicated that low levels of sucrose in plant organs reduces their ingestion by insect pests (Farrell 1977), then reinforces the resistance through inefficient assimilation. Similarly, past research supports the view that factors affecting food assimilation include low nutrient concentration (e.g., sucrose) in plant organs (Kennedy and Schaefers 1975). Sucrose was the highest soluble sugar content found in barley susceptible genotypes to aphid (Corcuera 1993). Also, Quiros et al. (1977) found significantly higher sucrose concentration in tomato susceptible plants to potato aphid *Macrosiphum euphorbiae* Thomas (Homoptera: Aphididae). The study did not establish a clear link between the total sugar and the resistance/susceptibility of the test entries. This result corroborates the work of MacFoy and Dabrowski (1984) who did not find any correlation between the total sugar and the resistance in some cowpea genotypes to *A. craccivora*.

Apart from sugar compounds, the biochemical analysis found a high proportion of some polyphenolic compounds from aglycones such as kaempferol and quercetin in the resistant mini-core accessions as well as in the resistant check TVu-801. This result suggests that these two polyphenols play significant roles in the resistance of cowpea genotypes to *Aphis craccivora,* confirming an antibiosis mechanism. This corroborates some findings which showed that the resistance in TVu-801 was an antibiotic effect (Singh et al. 1982, 1984; Jackai and Singh 1988; Ofuya 1993, 1997; Jackai and Adalla 1997). Aphid resistance in this variety was attributed to its phenolic or flavonoid contents (Ofuya 1997). Kaempferol and quercetin were the main phenolic compounds found in the resistant mini-core genotypes in higher quantity than in susceptible genotypes, therefore they surely have reinforced the resistance in these accessions. This is in agreement with the finding of Lattanzio et al. (2000), which reported that quercetin and kaempferol are major flavonoid components of cultivated cowpea where the proportion is higher in aphid-resistant genotypes than in aphid-susceptible ones. Similarly, it was reported that the flavonoid quercetin possesses a good inhibitory rate to aphid reproduction (Lattanzio et al. 2000). According to past studies, the antibiosis mechanism mediated by cowpea allelochemicals is governed by a single dominant gene (Singh and Ntare 1985; Pathak 1988; Van Emden 1991). In contrast, other resistant genotypes such as TVu-1583 showed high levels of sucrose. This demonstrates that the resistance in this genotype relies on its high phenolic content only.

The study reported here confirms the resistance status of TVu-801 to *A. craccivora* (Ofuya 1997) and also the susceptibility of TVx-3236 as reported in some past studies (Bata et al. 1987; Souleymane et al. 2013). A positive correlation was observed between aphid susceptibility and sucrose content in cowpea.

In view of our findings, it appears that aphid resistance in cowpea mini-core genotypes relies on two major factors. The first factor is low sucrose content in the host plant, and the second factor is the high content in phenolic aglycones, namely, kaempferol and quercetin. Cowpea mini-core genotypes showing high levels of the phenolic compounds (e.g., TVu-6464, TVu-15445, and TVu-1583) and a low level of sucrose (e.g., TVu-6464 and TVu-15445) were observed to record low levels of damage during the screening tests. They also resulted in high mortality of aphids during laboratory bioassay. The very good level of resistance to *A. craccivora* in these identified genotypes suggests that the mechanism of resistance is antibiosis mediated by the cited factors.

## Conclusions

This study identified three cowpea mini-core germplasm genotypes, TVu-6464, TVu-1583, and TVu-15445, with good levels of resistance to *Aphis craccivora*. The biochemical characterization revealed that the resistance mechanism involved in these genotypes was mediated by the high content of two phenolic aglycone (kaempferol and quercetin) and low content of one sugar metabolite (namely the sucrose). The mini core genotypes identified with good resistance are potential sources of aphid resistance genes and can be used in the cowpea breeding program to improve the crop’s performance in *Aphis craccivora* prone farmers’ fields. Moreover, data generated in this study can be used in genome-wide association studies to identify QTLs associated with aphid resistance.
